# Expanding the bioluminescent reporter toolkit for plant science with NanoLUC

**DOI:** 10.1186/s13007-019-0454-4

**Published:** 2019-07-08

**Authors:** Uriel Urquiza-García, Andrew J. Millar

**Affiliations:** 1 SynthSys and School of Biological Sciences, University of Edinburgh, C. H. Waddington Building, King’s Buildings, Max Born Crescent, Edinburgh, EH9 3BF Scotland, UK; 2 Institute for Molecular Plant Sciences, University of Edinburgh, D. Rutherford Building, King’s Buildings, Edinburgh, EH9 3BF UK

**Keywords:** Chronobiology, Reporter gene, Luciferase, Arabidopsis, Synthetic biology, Gene expression, Protein dynamics, Bioluminescence

## Abstract

**Background:**

Protein data over circadian time scale is scarce for clock transcription factors. Further work in this direction is required for refining quantitative clock models. However, gathering highly resolved dynamics of low-abundance transcription factors has been a major challenge in the field. In this work we provide a new tool that could help this major issue. Bioluminescence is an important tool for gathering data on circadian gene expression. It allows data collection over extended time periods for low signal levels, thanks to a large signal-to-noise ratio. However, the main reporter so far used, firefly luciferase (FLUC), presents some disadvantages for reporting total protein levels. For example, the rapid, post-translational inactivation of this luciferase will result in underestimation of protein numbers. A more stable reporter protein could in principle tackle this issue. We noticed that NanoLUC might fill this gap, given its reported brightness and the stability of both enzyme and substrate. However, no data in plant systems on the circadian time scale had been reported.

**Results:**

We tested NanoLUC activity under different scenarios that will be important for generating highly quantitative data. These include enzyme purification for calibration curves, expression in transient plant systems, stable transgenic plants and *in planta* time series over circadian time scales. Furthermore, we show that the difference in substrate use between firefly luciferase and NanoLUC allows tracking of two different reporters from the same samples. We show this by exploring the impact of a BOAp:BOA-NanoLUC construct transformed into a Col-0 CCA1p:FLUC background.

**Conclusions:**

We concluded that NanoLUC reporters are compatible with established instrumentation and protocols for firefly luciferase. Overall, our results provide guidelines for researchers gathering dynamic protein data over different time scales and experimental setups.

## Background

Bioluminescent reporter genes (luciferase genes, abbreviated *LUC*) have offered a window into the dynamic regulation of metabolite levels, gene expression, protein accumulation and cell tracking in vivo, in diverse biological systems, for over 30 years [1, 2]. Long-term studies of 24-h rhythms driven by the biological clock have particularly benefited from luciferase technologies. The underlying circadian clock mechanisms are often sensitive to light, so fluorescent reporters that require excitation light would artefactually interfere with this system. Luciferase (FLUC) from the North American firefly *Photinus pyralis* has been widely used in plant chronobiology and in other studies of dynamic gene regulation [3–9]. 24 h Oscillations of light emission are observed when the substrate luciferin is provided externally and can be quantified by imaging or by luminometry over several days. Multiple derivative versions (termed *LUC*+, *LUC2* and others) have modified the native FLUC protein’s peroxisomal targeting, potential transcript splicing, codon usage and light output, making the reporter assays more robust. However, the rhythmic assays depend upon the short half-life of the reporter to reveal both decreasing and increasing gene expression rate from the sequences that control the *FLUC* reporter gene, and this instability depends, in turn, on the complex biochemistry of the FLUC protein.

In plants and algae, FLUC enzyme activity in vivo is destabilised in part post-translationally, in the presence of luciferin [10]. Newly-synthesised protein is thought to be active, but the cells can also accumulate inactive FLUC protein over time. This regulation of reporter activity can be an unattractive, complicating factor in experimental design, especially where FLUC is used in a translational fusion to report protein abundance. Results from fusions of FLUC to other, unstable proteins suggest that FLUC degradation can be controlled by the fusion partner [10, 11], but for stable fusions, the concern remains that FLUC activity in vivo could fall long before the fusion protein was degraded. In contrast, FLUC protein degradation is faster and contributes more to reporter dynamics in mammalian cells tested at higher temperatures [12], where destabilising protein tags have been used to report still faster dynamics, in transcriptional bursting [13]. A stably-active luciferase as reporter fusion protein would facilitate longitudinal studies of dynamic protein stability *in planta*. The engineered Nano Luciferase (NanoLUC, NLUC) [14] offered several advantages for this application.

NanoLUC is a small protein (19.2 kDa, compared to 59 kDa FLUC), originally from the deep-sea shrimp *Oplophorus gracilirostris*, engineered for high stability (t_1/2_ = 11.5 days at 37 °C) and codon optimised for expression in mammalian cells [14]. The enzyme only requires O_2_ and substrate to produce a strong blue light signal, whereas FLUC also depends upon endogenous ATP and Mg^++^, which can fluctuate in a circadian manner [15]. A recently-developed substrate for NanoLUC is Furimazine, a more stable analogue of the naturally-occurring substrate coelenterazine [16]. Instability of luciferin substrate can also be a concern in long-term FLUC assays as it can introduce an artefactual trend in circadian data, which can complicate period estimation [17].

NanoLUC shows stable light emission around 22 °C and stable activity in the physiological pH for Arabidopsis (7.1 in nucleus, 7.2 in cytosol [18]), whereas FLUC activity is sensitive to physiological changes in temperature and pH [14, 16]. The issues noted above are less relevant for well-controlled, in vitro LUC assays of plant extracts. As a final benefit, NanoLUC signal was reported to be three orders of magnitude brighter than FLUC [14], increasing the sensitivity of detection for low-abundance proteins such as regulators of the circadian clock both in vivo and in vitro. However, no reports had tested NanoLUC as a dynamic reporter protein in plants [19].

We tested NanoLUC in transgenic Arabidopsis plants, using a translational fusion to the rhythmic transcription factor BROTHER OF LUX ARRHYTHMO (BOA, also known as NOX) as a test case. The evening-expressed *BOA* gene was shown to function together with its homologue *LUX ARRYTHMO* (*LUX*) to regulate the expression of the morning-expressed gene *PSEUDO RESPONSE REGULATOR 9* (*PRR9*), in an *EARLY FLOWERING 3* (*ELF3*)-dependent manner [20]. In addition, overexpression of BOA has been shown to alter the period of the clock in constant light conditions [21]. A detailed mathematical model F2014 included BOA protein as a component of the Evening Complex, a rhythmic transcriptional repressor [22]. Published data on BOA protein accumulation is extremely limited [21] and further evidence of rhythmic BOA protein accumulation will help to constrain future models. To this end, we purify NanoLUC protein and test its stability in vitro, demonstrate the activity of NanoLUC in Arabidopsis extracts, test the sensitivity and linearity of the in vitro assay, and test in vivo reporter activity in protoplasts and transgenic plants. Comparing the results from translational fusions of BOA-NanoLUC and BOA-FLUC in transgenic plants with a simple mathematical model suggested that NanoLUC was more stable than FLUC in vivo. Our results show that NanoLUC is a useful reporter of dynamic protein regulation in plants over the circadian time scale, with potentially significant advantages for in vitro assays. Its performance in vivo is promising and merits further investigation.

## Results

The expected Codon Adaptation Index for expression of NanoLUC in *Arabidopsis* is remarkably good, with eCAI = 0.737 (p < 0.05) calculated by E-CAI [23], on a scale where 1 is a sequence with the most common codons in the organism. NanoLUC was codon-optimised for mammalian expression with an eCAI = 0.803 (p < 0.05), in Human. The commercially-available sequence was therefore appropriate to use without further optimisation.

### Activity and purification of NanoLUC variants

**Fig. 1:**
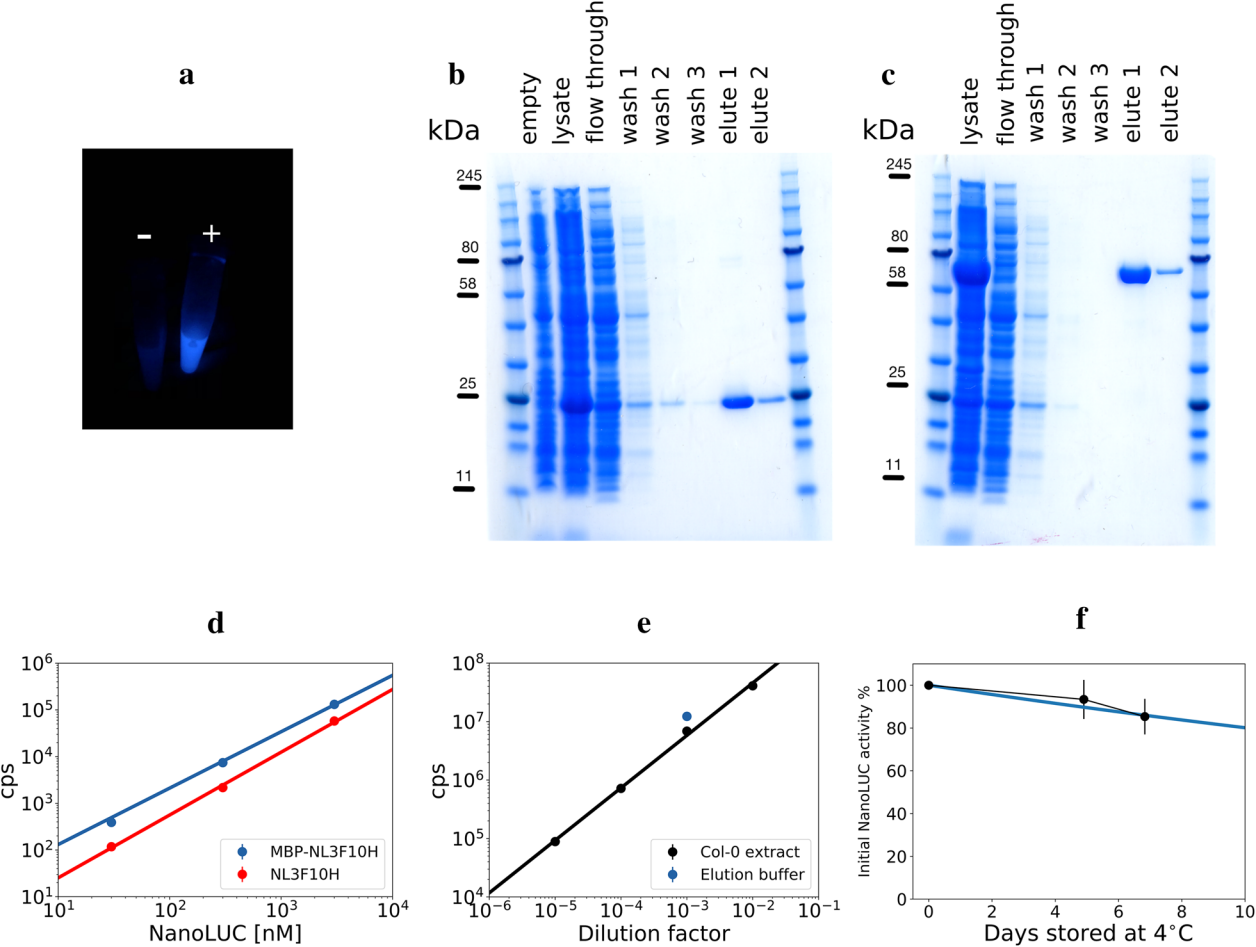
Recombinant expression of NanoLUC variants and characterisation. **a** 1.5 ml microcentrifuge tubes containing *E.coli* Rosetta 2 BL21 pLyS pET28a:NanoLUC cells induced with IPTG (+) or without IPTG (-) after being incubated for 6 h at 30 °C 200 r.p.m. Photo taken with a standard mobile phone camera to exemplify NanoLUC brightness. **b** Purification of NanoLUC-3× FLAG-10× His (NL3F10H, ~ 23.53 kDa) by IMACS, wash steps done with wash buffer, elution 1 and 2, performed with 200 µl of elution buffer. **c** Purification of Maltose-Binding-Protein-NanoLUC-3× FLAG-10× His (MBP-NL3F10H, ~ 66.83 kDa) by IMACS. Purification described in methods. 10 µl of each purification fraction was loaded into a NuPAGE 4-12% Bis–Tris (Invitrogen) and run at 200 V for 40 min. **d** Linearity of NanoLUC invitro assay. Protein was quantified by linearized Bradford assay and adjusted to 10 mM. Serial dilutions were generated for purified NL3F10H or MBP-NL3F10H shown in B and C, enzymatic assays were performed as in F. **e** Effect of Col-0 plant extract on the activity of NanoLUC. Black circles, serial dilutions of purified MBP-NL3F10H in plant extract of 21 days old Col-0 plants. The extract was obtained by liquid nitrogen freezing and grinding in Tissue lyser. Grinded tissue was resuspended in BSII buffer to 0.4 gFW ml^−1^. Blue circle, 1:1000 dilution of MBP-NL3F10H in BSII buffer. **f** Stability of NanoLUC. MBP-NL3F10H was stored at 4 °C in Elution buffer (250 mM NaCl, 50 mM NaH_2_PO_4_ pH 8.0 NaOH adjusted, EB). The purified enzyme was diluted 1:1000 in Elution buffer and mixed with 100 µl of 1:50 furimazine:NanoGlow buffer in a 96-well flat black plate (Lumitrac, Greiner). Samples were incubated for 10 min at 21 °C in a Tristar luminometer plate reader. Solid blue line fitted exponential decay model results in a half-life of ~ 37 days. Circles, mean of three biological replicates, error bars S.E.M

In order to verify the activity of NanoLUC (NL), the coding sequence provided by Promega was cloned into pET28a(+), for expression in *E. coli* BL21 Rosetta2. After 6 h of induction with 1 mM IPTG at 30 °C, cells were harvested by centrifugation, re-suspended in 1 ml of Phosphate Buffered Saline (PBS) and 1 µl of Furimazine (at the stock concentration). A culture of Cells expressing NL showed bioluminescence visible to the naked eye, while cells carrying the empty pET28a(+) vector did not (Fig. 1a). We created two NL derivatives. One carried a C-terminus 3× FLAG-10× His, which allows purification by ion metal affinity purification (IMAC) using a Ni–NTA matrix as a stationary phase, thanks to the 10× His region. We also created a MBP-NL-3F10H (Maltose Binding Protein, MBP) for testing the effect of fusing proteins to the N-terminus of NL. The MBP derivative used brings 6× His on the N-terminus, so this NanoLUC presents 16× His.

The resulting constructs were introduced into *E. coli* BL21 Rosetta 2 pLysS for expression and purification. The three versions NL, NL3F10H and MBP-NL3F10H presented high expression levels at 30 °C and remained soluble after centrifugation for 20 min at 20,000×*g* (Fig. 1b, c, lysate lane). We performed an IMAC purification using Ni–NTA agarose beads using the manufacturers standard protocol, resulting in successful isolation of the fusion proteins. As expected, the NL original variant does not bind to Ni–NTA beads and can be removed after two washes with washing buffer (WB), data not shown. The NL3F10H version did bind to the Ni–NTA beads and can be eluted with the 200 mM Imidazole present in the Elution Buffer (EB) (Fig. 1b). As expected, MBP-NL3F10H migrates significantly more slowly relative to NL3F10H in PAGE gels (Fig. 1c), and also resulted in a homogenous preparation. This shows that purification of NL can be performed in a straight forward way for several uses, including generation of calibration curves for inferring NanoLUC concentration in plant extracts.

We then compared the activity of NL3F10 to MBP-NL3F10H in order to test for detrimental effects on activity from adding the Maltose Binding Protein as a N-terminal tag, a fusion equivalent to using NL3F10H as a C-terminal tag to our target clock transcription factors. We purified NL3F10H and MPB-NL3F10H variants as before. A dialysis step was added in order to remove the excess of Imidazole used for eluting the protein from the Ni–NTA beads, which can interfere with the Bradford protein assay. The two variants were quantified for protein content using a linearized version of the Bradford assay [24]. We explored the effect of serial dilutions on the activity of the variants, expecting a proportional drop in activity after each dilution. We did not observe any strong departure from linearity. However, we observed lower enzymatic activity for the NL3F10H variant compared to the MBP-NL3F10H in the tested conditions (Fig. 1d). This difference could be explained by a stabilisation or folding enhancement from the MBP tag, helping to recover more active NLUC [25]. The MBP tag also adds the advantage of a possible secondary purification step, using an Amylose column which can be bound by MBP.

Then we explored whether plant extracts could inhibit MBP-NL3F10H activity, by making serial dilutions of MBP-NL3F10H in a crude extract of 21-day-old Col-0 rosettes. The activity of the NanoLUC preparation was such that a 1 × 10^−3^ dilution was required to avoid saturation of the plate reader (Fig. 1e). The luminescent signal decreased consistently by an order of magnitude after each dilution in plant extract, without progressive inhibition. However, NanoLUC had slightly higher activity in a dilution with Elution Buffer than in the dilutions with plant extract, so a minor inhibitory effect is possible. This experiment highlights the importance of using representative assay conditions for calibration curves that will be eventually used to infer the concentration of a fusion protein.

Working with an enzyme as a calibrator can be problematic if the activity is very unstable. If the enzyme decays fast in the calibrating preparations, this will result in an overestimation of the tagged protein concentration in freshly prepared plant extracts. Therefore, we did a simple experiment to test NanoLUC stability and derived a first order kinetic decay model for the enzyme activity. The model can then be used for correcting quantifications if the enzyme is not used immediately after purification. Our results show that MBP-NL3F10H retains more than 95% of activity before 3 days and more than 80% after 1 week at 4 °C. Linear regression on the log-transformed time series resulted in a half-life of 37.2 days (Fig. 1f). Therefore, MBP-NL3F10H is a stable enzyme which can be used to generate calibration curves in plant extracts, for later quantification of NanoLUC-tagged fusion proteins.

### Extending pGWB with a versatile NanoLUC tag

The well-established and flexible pGWB vector series is a good choice for protein functional studies in plants [26, 27]. This collection of vectors uses a tail-to-tail construct: marker design, which might help to mitigate unintended effects of the selection marker on the expression of the synthetic construct. Secondly, the promoter on the selection marker is a NOPALINE SYNTHASE (NOS) promoter rather than a CaMV35S promoter. It has been shown that the CaMV35S promoter can result in secondary effects on the expression of nearby genes, possibly affecting the expression of the construct of interest [28]. Also, an extensive library of Gateway clones exists for plant systems therefore easing the use of NanoLUC without the need to re-clone these DNA parts. Finally, they present a good diversity of selection markers (Kanamycin, Hygromycin, BASTA and Tunicamycin). This is an important feature as many single and higher-order Arabidopsis mutants already carry selection markers, yet complementation of these mutants is crucial to test the functionality of the fusion proteins in vivo.

**Fig. 2:**
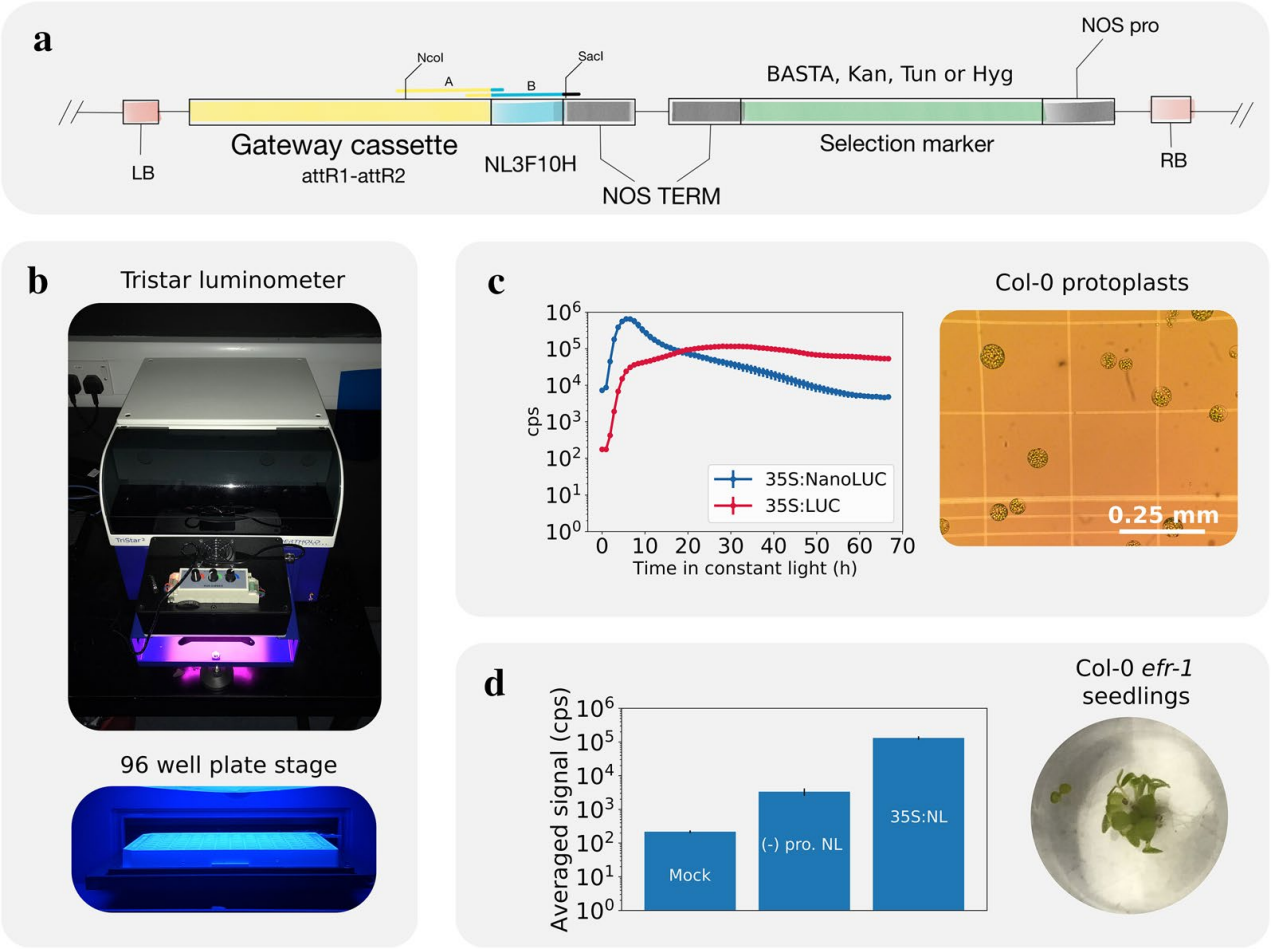
Generation of NanoLUC vectors and characterisation in transient expression systems. **a** Structure of pGWB vectors extended for NL3F10H translational fusions. The Gateway NL3F10H cassette was generated by Gibson Assembly of PCR products A and B, designed with overlapping regions between them and pGWB601 vector. The vector was digested with Nco/SacI restriction enzymes to insert the new cassette. T-DNA Left Border (LB) and right border (RB). NOPALINE SYNTHASE promoter and terminator (NOS pro, NOS Term). Gateway cassettes of type attR1-attR2 can be used as destination in a LR Gateway cloning reaction. The pGWB vectors have a common Scp^r^ marker for selection in bacteria, with a pPZP vector backbone. The variants used here were pGW401 (Kan^r^), pGWB501 (Hyg^r^), pGWB601 (BASTA^r^) and pGWB701 (Tun^r^). NL3F10H was introduced in each vector by NcoI/SacI sub-cloning. **b** Plate reader set up for measuring bioluminescence signal on a 96-well plate format using a Tristar luminometer plate reader (Berthold). Light was a mix of red:blue 2:1 LEDs, total intensity 50 µmol m^−2^ s^−1^. **c** Activity of pGWB601::35S:NL3F10H and pGWB635::35S:LUC in protoplasts over several days. Protoplasts were isolated and transformed with equimolar amount of plasmid by the method of Hanse et al. [29] and followed in a 96-well black plate for 3 days in constant light at 21 °C. Furimazine was used as substrate for NL3F10H 1:50 Furimazine:W5 buffer and for LUC 5 mM luciferin. Mean of three technical replicates error bars show S.E.M. **d** Transient transformation of *Arabidopsis efr*-*1* seedlings with NanoLUC. Seedlings were transformed by the AGROBEST method using either promoterless pGWB601NL3F10H or pGWB601::35S:NL3F10H. Seedlings were transferred to a 96-well flat white plate with 50 µl of 1:50 Furimazine:0.01% Triton X-100 and tracked for 48 h in constant light. Bars average signal over the time interval for a single pool of 10 seedlings. Error bars S.D

We used pGWB601 as a base vector and introduced NL3F10H by Gibson Assembly. This resulted in the new pGWB601NL3F10H, with BASTA resistance as a selection marker for plant transformation. This vector can then be used for building C-terminal translational fusions with NL3F10H by Gateway cloning. The new cassette was sub-cloned into pGWB401, pGWB501 and pGWB701, resulting in a collection of vectors with different plant selection markers (Fig. 2a).

### Testing NanoLUC activity in Arabidopsis

In order to test the activity of NanoLUC in Arabidopsis, we cloned the CaMV35S (35S) promoter from the pCambia1305.1 vector into a pDONR221 vector for subsequent recombination into vectors pGWB601NL3F10 and pGWB635. We used the latter vectors using 35S:LUC+ (an FLUC variant) constructs as positive control helping to rule out problems with either the 35S sequence or transformation methods, either in protoplasts or plants. We performed a transient expression experiment with protoplast from 4-weeks old Col-0 plants, with vectors containing 35S:NL3F10H and 35S:LUC+ constructs as described [29]. We used this protocol because it offered the possibility of tracking protoplast expression signals over circadian time scales. We followed the signal for 3 days in constant light conditions at 21 °C, in a modified luminometer plate reader (Fig. 2b). These results show that NL3F10H is translated into active enzyme in protoplasts. Interestingly the dynamics of the two constructs differed (Fig. 2c). 35S:NL3F10H expression drove transient expression that decayed from 9 h, whereas signal from the LUC+ construct peaked at 30 h and persisted for > 65 h. It is possible that the strong over-expression of NL3F10H resulted in greater substrate depletion compared to the LUC+ assay, however we did not further explore this hypothesis. In addition to protoplasts, we also tested NanoLUC using the AGROBEST method, which allows transient transformation of *Arabidopsis* seedlings by Agrobacterium [30]. Four days after germination Col-0 *efr*-*1* seedlings were co-incubated with *Agrobacterium* ABI strains, either carrying a promoterless NL3F10H vector or the 35S:NL3F10H construct. *Agrobacterium* without binary vectors was used as mock control. The *efr*-*1* mutation sensitizes *Arabidopsis* which otherwise is recalcitrant to *Agrobacterium* transformation by leaf infiltration. We observed strong bioluminescence in the 35S:NLF310H signal compared to the controls. Signal from the promoterless pGWB601:NL3F10H vector was > 50-fold lower than that driven by 35S but still ~ tenfold above the mock infiltration (Fig. 2d). The data indicated that the new vectors are competent for agro-mediated transformation in *Arabidopsis*.

### Overexpressing lines for optimisation of NanoLUC quantification in plant extracts

**Fig. 3:**
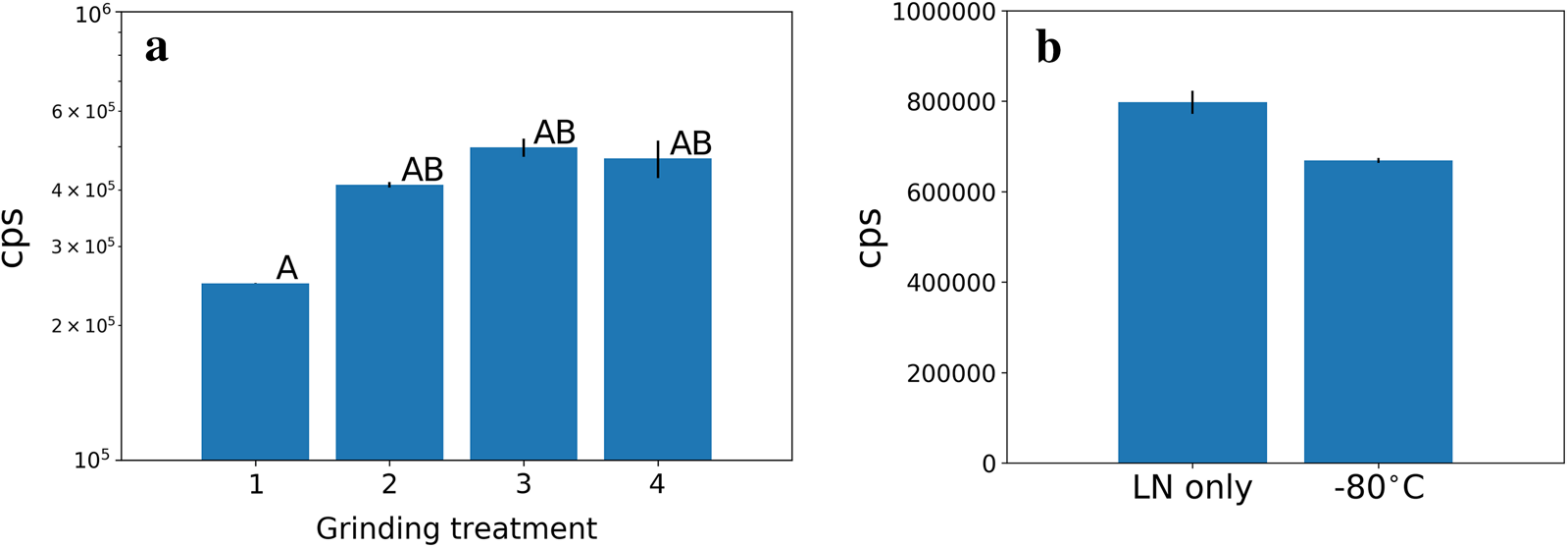
Extraction of NL3F10H from tissue of stable transgenic lines. **a** Comparison of extraction method, (1) manual grinding (mean = 12.42, std = 0.001), (2) treated once with liquid nitrogen and grinded in a Tissue lyser (Qiagen) (mean = 12.95, SD = 0.02). (3) Second round of grinding in Tissue lyser (mean = 13.1, SD = 0.06). (4) Third round of grinding of Tissue lyser (mean = 13.05, std = 0.13). (A) *F*-value (3,4) = 16.82, *p* value = 0.009. (B) *F*-*value*(2,3) = 1.17, p-value = 0.41. **b** Testing activity decay after storing tissue at − 80 °C (ln(mean) = 13.58, SD = 0.045) for 2 days compared to liquid nitrogen (LN) (mean = 13.41, SD = 0.01). 10 µl of plant extract mixed with 190 µl of BSII buffer then loaded in a 96-well black plate. Bars represent the mean three biological replicates with error bars representing S.E.M. *t*-Value [2] = 3.7, p-value = 0.065. All statistical tests performed with ln-transformed data

We transformed Col-0 plants by floral dipping to generate a collection of stable transgenic lines using the pGWB601:35S:NL3F10H vector. These lines can be used as positive controls of NanoLUC activity, and as negative controls if NanoLUC-3× Flag-10× His is later used as an epitope tag to other proteins, for example in Chip-seq or Co-IP assays. Using BASTA resistance as a marker we performed segregation analysis, selecting lines that present single insertions judged by Mendelian ratios (3:1). Then, we evaluated the effect on NanoLUC activity when preparing plant extracts either by manual grinding or by several rounds of tissue disruption in a bead beater (Tissue Lyser, Qiagen). We observed that manual grinding with a pestle is less efficient compared to one or more rounds of mechanical extraction. After the first round of tissue grinding with Tissue Lyser, no further NanoLUC activity was extracted, at least not statistically significant (Fig. 3a). Nonetheless, we suggest two rounds of grinding. The grinding experiments were performed with plants frozen with liquid nitrogen. However, in many experiments, storing samples at − 80 °C is a usual step (e.g. RNA isolation). Therefore, we tested this situation by flash freezing 2-weeks old seedlings expressing NanoLUC then storing them at − 80 °C overnight. We observed a small decay in activity after storage, compared to processing immediately after flash freezing (Fig. 3b). The decay is just barely not statistically significant, nonetheless we suggest that samples should be processed on the same day or stored in liquid nitrogen when time series are generated, unless further research optimises storage conditions.

### BOA-NL3F10H and BOA-LUC present different dynamics

Our experimental results show that NanoLUC is active in plants in several experimental setups. However, one of the reasons we are using it is the reported stability and higher brightness reported relative to FLUC, with NanoLUC being 1000× brighter than FLUC. The NanoLUC stability might in principle facilitate faithful tracking of protein levels in vivo. Therefore, we tested the dynamics of fusion to *BROTHER OF LUX ARRHYTHMO* (*BOA*), also known as NOX. We cloned the genomic region of *BOAp:BOA* and created a translational fusion with NL3F10H using the pGWB601NL3F10 vector, then transformed Col-0 plants carrying the rhythmic CCA1p:LUC+ reporter, in order to test the effect of BOA-NL3F10 on the clock. BOA has been suggested to be an activator of *CCA1* transcription, which should be detectable in the Col-0 CCA1p:LUC+ background [21]. We also created a BOAp:BOA-LUC+ construct and transformed the Col-0 genotype, to compare NL and FLUC reporters of BOA.

**Fig. 4:**
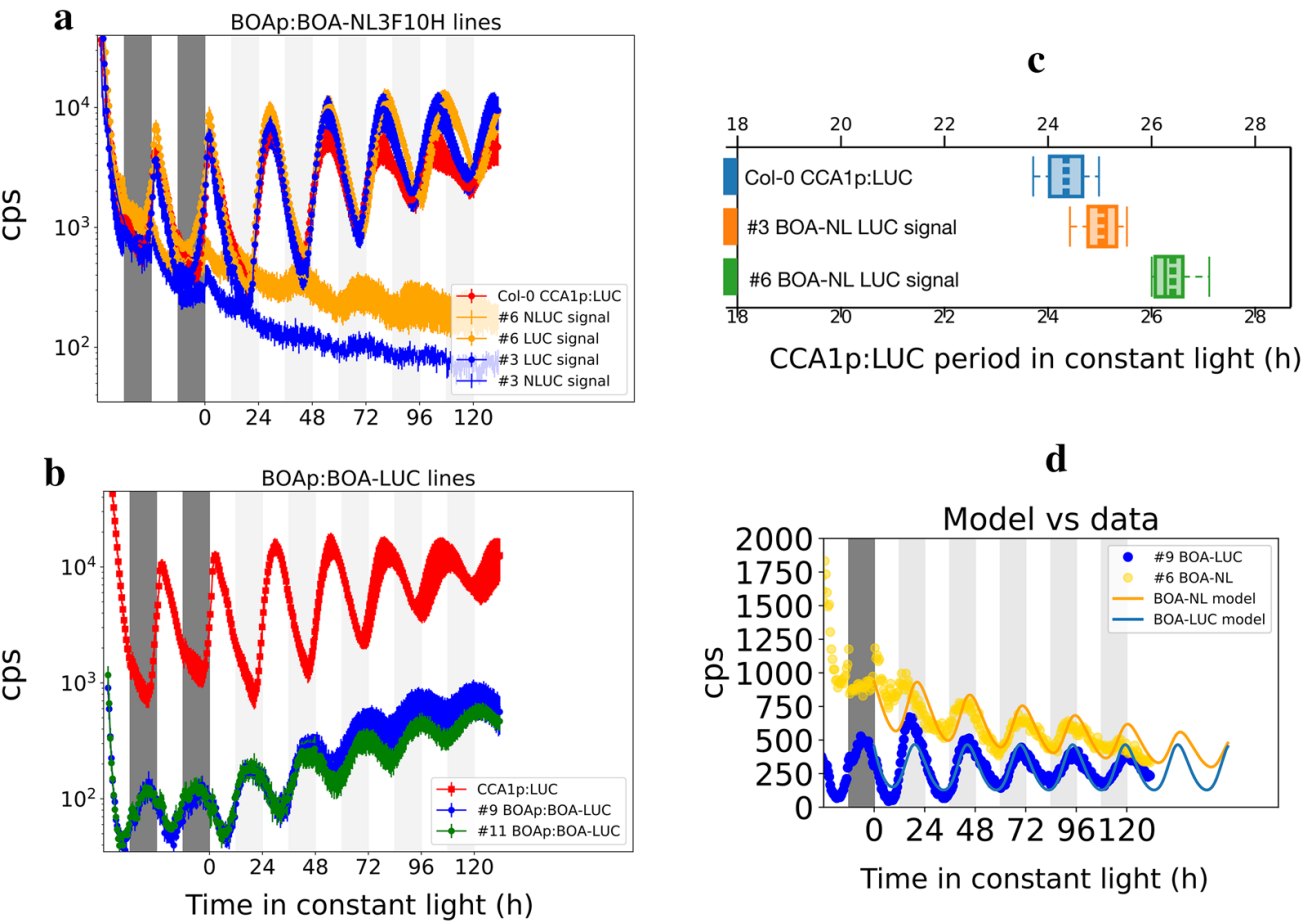
Dynamics BOA protein using NanoLUC and LUC reporters. **a** Dynamics in Col-0 CCA1p:LUC BOAp:BOA-NL3F10H using two independent T3 lines, The signal of CCA1p:LUC (triangle) and BOA-NL3F10H (squares) were measured in different wells, the control is shown in red. **b** Dynamics of BOAp:BOA-LUC in Col-0. Seedlings were grown for 7 days at 100 µmol m^2^ s^−1^ white light 12L:12D 21 °C. Then transferred for 2 days into 50 µmol m^2^ s^−1^ monochromatic red and blue LEDs 2:1, before quantification started using a Tristar luminometer plate reader every 30 min with 1.5 s of signal integration. The traces are means of 4 replicates. Error bars represent S.E.M. **c** BOA-NL impacts the period of CCA1p:LUC. X-axis period estimates for CCA1p:LUC time-series from A determined by FTT-NLS from Biodare2. **d** Inferring species stability by modelling. The experimental data is represented by dotted lines, BOA-NL (blue) and BOA-LUC (yellow). Model traces are represented by solid lines of respective colours. The inference of model parameters was performed with the brightest traces for both BOA-NL and BOA-LUC. The inferred parameters are presented in Table 1

We performed a plate reader counting experiment with two independent, transgenic plant lines for each construct, using as positive control for phase and period the Col-0 CCA1p:LUC+ (Fig. 4). The BOAp:BOA-NL3F10 signal was rhythmic but with a low amplitude in constant light (Fig. 4a). However we observed a correlation between the NanoLUC signal level and increased period of the CCA1p:LUC reporter in the same transgenic lines (Fig. 4c). This is consistent with the previous report that BOA-OX lines present longer periods relative to the WT [21], and suggests that the BOA-NL3F10H fusion is biologically active. In contrast, signals from BOAp:BOA-FLUC plants had a high amplitude and reflected the night-peaking expression pattern previously reported for the *BOA* transcript by qRT-PCR [21], with the lowest levels at mid-day under entrainment conditions (Fig. 4b). We observed that BOA expression presented rhythmic circadian oscillations in constant light, and CCA1p:LUC and BOA-FLUC had an antiphase relationship. The signal for two representative BOA-FLUC lines was lower than CCA1p:LUC, potentially consistent with low abundance of BOA protein [21]. Figure 4d compares the strongly rhythmic BOA-FLUC signals (replotted from Fig. 4b) to the results from BOAp:BOA-NL3F10H (from Fig. 4a). In addition to different reporter protein, effects local to the transgene insertion sites might contribute to the difference in absolute expression levels.

One salient difference between the BOA-NL3F10H and BOA-FLUC lines is the reporter sequence, and this work was originally motivated to explore if the relatively stable NanoLUC protein and enzyme activity could provide a better approximation to native protein dynamics compared to FLUC. We used the following simple mathematical model to test the potential effect of protein stability, by estimating the decay constants of FLUC and NanoLUC from the in vivo time series:

$$\frac{dcBNL}{dt} = k_{1} \exp \left\{ {\sin \left( {\frac{2\pi }{25}t + 10} \right)} \right\} - \left( {k_{2} + k_{3} } \right)cBNL$$

$$\frac{dcBFLUC}{dt} = k_{1} \exp \left\{ {\sin \left( {\frac{2\pi }{25}t + 10} \right)} \right\} - \left( {k_{2} + k_{4} } \right)cBFLUC$$

where *cBNL* and *cBFLUC* are the state variables for BOA-NL3F10H and BOA-FLUC. *k*_1_ is the translation rate, which is identical between the two constructs; *k*_2_ is the degradation rate of BOA protein, which affects both constructs; *k*_3_ is the decay rate of NanoLUC enzyme activity and *k*_4_ the decay rate of FLUC enzyme activity. *k*_1_, *k*_2_ and *k*_4_ were adjusted manually to match the experimental data (Fig. 4d and Table 1). The decay rate of NanoLUC activity was estimated using the in vitro decay rate measured at 4 °C (Fig. 1f), adjusted to the 21 °C conditions of the plate reader experiment by assuming Q_10_ = 2.5. Although this coarse estimate was likely a lower bound, this very slow decay rate of NanoLUC activity nonetheless allowed the model to fit the data. Modelling results consistent with the data suggested that BOA protein had a half-life of 8.66 h. Therefore, BOA protein was more stable than FLUC activity, with an estimated half-life 4.6 h (close to a previously-determined value [31]). The model suggested that the additional effect of the slow, NanoLUC degradation on reporter signal was small, whereas the greater instability of FLUC in vivo dominated the reporter dynamics and resulted in high-amplitude rhythms. This result supports the use of NanoLUC as a reporter for protein dynamics.

**Table 1:** Model parameters, assuming only decay constants *k*_3_ (NanoLUC) and *k*_4_ (LUC) differ between reporters

Parameter	Value	Units	Source	Process
*k*_1_	50	cps h^−1^	Fitted	Translation
*k*_2_	0.8	h^−1^	Fitted	BOA decay
*k*_3_	0.0121	h^−1^	Experimental	NanoLUC decay
*k*_4_	0.15	h^−1^	Fitted	LUC decay
*a*	0.004	h^−1^	Fitted	Furimazine decay

Fitting the parameters show that is possible to replicate the signal in Fig. 4d. NanoLUC decay rate was obtained by scaling the experimentally determined rate assuming a Q_10_ = 2.5 as plate reader experiments were conducted at 21 °C. The NL decay trend could possibly be explained by decay in the furimazine concentration, for which a exponential decay was introduced. Troein et al. [39] estimated a LUC decay constant of ~ 0.183 h^−1^ in *Ostreococcus tauri*

## Discussion and conclusions

The use of tagged proteins with general epitopes e.g. GFP, FLAG or 10× His has been a powerful approach for studying the protein components of the *Arabidopsis* circadian oscillator. Thanks to these transgenic lines, several aspects of clock protein regulation have been investigated, including localisation, binding sites in the genome by ChIP-seq, and protein–protein interactions by Co-IP or more recently, affinity purification followed by mass spectrometry [32–37]. We therefore explored the use of NanoLUC as a new protein tag for circadian biology in plants.

Our results showed that it is possible to work with NanoLUC using the infrastructure developed for tracking FLUC bioluminescence. The protocols above support calibrated, in vitro assays that could provide absolute quantification of NanoLUC in plant extracts. The NanoLUC protein proved highly active in both transient expression and stable transgenic plants, with convenient assay protocols, and thus provides a complementary reporter to FLUC in assays from extracts. Our 35S:NL3F10H plants will provide controls for such studies. Both FLUC and NanoLUC require specific substrates, respectively d-luciferin and coelenterazine or the commercial alternative furimazine, so their cost will be a factor in assay adoption.

We tested NanoLUC in a real scenario by generating new protein data for BOA, where we observed a discrepancy between the test NanoLUC fusion and an FLUC control. Both constructs were generated from the same pDONR221:BOApBOA-no-stop vector, therefore the only differences should be the reporter tag and transgene insertion sites. BOAp:BOA-FLUC presented clear oscillations in light emission, whereas the equivalent BOAp:BOA-NanoLUC construct drove lower-amplitude rhythms. One possible cause of this difference in behaviour is a difference in the stability of FLUC and NanoLUC. The instability of FLUC activity was advantageous in its original application, tracking RNA abundance rhythms, where the rate of new reporter synthesis was the critical variable and the total protein pool was not directly relevant [3]. We wanted to exploit NanoLUC because its stability might better represent those total protein levels, especially for more stable proteins.

The mathematical exercise we presented shows that the difference in reporter dynamics can be accounted for by a difference in stability between FLUC and NanoLUC. Combining the experimentally data and mathematical model, we estimated a NanoLUC half-life of ~ 57 h, such that more rapid degradation of the BOA protein moiety (half-life 8.66 h) would dominate the fusion reporter dynamics. Our half-life estimates for FLUC (4.6 h) is consistent with previous estimates in the alga *O. tauri* (3.8 h) [9]. In mammalian cells measured FLUC half-life was ~ 2 h at 37 °C, though extrapolating to the half-life at 21 °C using Q_10_ = 2.5 yields an estimate of ~ 5 h [12], similar to ours. The greater stability of NanoLUC suggests that it might poorly report dynamic transcriptional regulation, or would give low-amplitude temporal profiles, because the stable reporter protein could not be destabilised by a fused clock protein as it can be in translational fusions. The destabilised NanoLUC-PEST version might be helpful in transcriptional fusion studies [14]. Further studies are clearly warranted, not only on stability but also on topics we have not addressed, such as the efficiency of furimazine permeation into seedlings. Nonetheless, we expect to see increasing use of NanoLUC for in vitro assays from plant samples, where it has great promise as a protein reporter, and potentially also for in vivo analysis.

## Methods

### Construction of plasmids and bacterial work using NanoLUC

Plasmids pNL1.1 and pNL1.2 with NanoLUC sequence were kindly provided by Promega Corporation (UK, Delta House, Southampton Science Park, Southampton, SO16 7NS) and propagated in *E. coli* DH5alpha (Invitrogen, ThermoFisher Scientific, Waltham, Massachusetts, US). The NanoLUC sequence was amplified, and cloned via a pET28a(+) (Novagen, Merck, Darmstadt, Germany) intermediate vector into a pUC19 vector carrying a 3× FLAG 10× His peptide as a C-terminal translational fusion, to form NanoLUC-3× FLAG-10× His (NL3F10H). The 3× FLAG-10His was chemically synthesised by Eurogentec (Seraing, Belgium). The tagged NanoLUC was amplified and cloned using Gibson assembly into pET28a(+) resulting in pET28a(+)::NL3F10H. A N-terminal tag comprising Maltose Binding Protein 6× His was fused to NanoLUC-3F10H, by amplifying the MBP-6× His from pMJ806 [38], resulting in pET28a(+)::MBP-NL3F10H. Each intermediate amplified step was sequence-verified after cloning, by Sanger sequencing (Genepool Edinburgh Genomics, Edinburgh, UK).

### NanoLUC purification protocol for calibration curves

The MBP-NL3F10H protein can be overexpressed and purified from *E. coli* using the following protocol:

Chemicals were obtained from (Sigma-Aldrich, Merck, Darmstadt, Germany) specified otherwise.

1. Three days before creating a NanoLUC calibration curve, transform the vector pET28a(+)::MBP-NanoLUC-3F10H into chemically competent *E. coli* cells of strain BL21 DE3 Rossetta2 pLysS (Novagen, Merck, Darmstadt, Germany), selecting transformants on solid LB media with 50 µg/ml Kanamycin (Kn^50^) and 34 µg/ml Chloramphenicol (Cm^34^). Incubate the plate over-night at 37 °C.
2. Pick a single colony and inoculate a 5 ml of LB broth with Kn^50^, Cm^34^ and incubate at 37 °C, 200 r.p.m. overnight.
3. Early next day, inoculate 100 ml of LB Kan^50^ Cm^34^ in a 0.5 l Erlenmeyer to a final O.D._600nm_ of 0.01. Incubate at 37 °C, 200 r.p.m. following the growth until the O.D._600nm_ has reached 0.5.
4. When the culture reached this point add IPTG to a final concentration of 1 mM and allow induction to proceed at 30 °C for 8 h.
5. Transfer the flask in ice-cold water for 10 min and then split the culture into two 50 ml polypropylene conical tubes. Harvest the cells by centrifugation at 4000×*g* and 4 °C for 15 min and discard the supernatant.
6. Resuspend the pellet in 10 ml ice-cold Lysis buffer (50 mM NaH_2_PO_4_, 300 mM NaCl, 10 mM Imidazole, pH 8.0 NaOH adjusted). At this point cells can be flash-frozen and stored at − 80 °C for later purification, this might impact the recovery of active enzyme. Therefore, we suggest further research on NanoLUC cryopreservation.
7. Disrupt the cells by sonication for 1 min, using Vibra-cell sonicator (Sonics & Materials Inc., Newton, Connecticut, US), with conditions 10 s on and 10 s off, 50% amplitude, in an ice bath.
8. Pass the crude lysate several times through a 25G (BD, Mississauga, Ontario, Canada) syringe needle to reduce lysate viscosity generated by genomic DNA.
9. Centrifuge 2 ml of lysate at 20,000×*g* and 4 °C for 20 min, which will eliminate unbroken cells and large debris.
10. Transfer 1 ml of supernatant to a 2 ml microfuge tube (Safelock, Eppendorf, Hamburg, Germany), and mix with 250 µl of Ni–NTA agarose (Qiagen, Hilden, Germany), agitate gently at 4 °C for 1 h.
11. Recover the Ni–NTA beads by centrifugation at 20,000×*g* and 4 °C for 1 min and transfer the supernatant to a clean 1.5 ml polypropylene tube for later analysis by SDS-PAGE.
12. Wash the Ni–NTA agarose beads three times with ice-chilled Washing Buffer (50 mM NaH_2_PO_4_, 300 mM NaCl, 20 mM Imidazole, pH 8.0 NaOH adjusted), centrifuging at 20,000×*g* and 4 °C each time.
13. Elute the MBP-NL-3F10H with 200 µl of Elution Buffer (50 mM NaH_2_PO_4_, 300 mM NaCl, 250 mM Imidazole, pH 8.0 NaOH adjusted).
14. Dialyse the elution fraction overnight using a 10,000 MW cut-off membrane at 4 °C in BII buffer (50 mM NaH_2_PO_4_, 300 mM NaCl, 20 mM pH 8.0 NaOH adjusted).
15. Determine the initial NanoLUC activity of the preparation in a 96-well flat white plate LUMITRAC (Greiner Bio-Once GmbH, Kremsmünster, Austria), using 30 µl of a 1 × 10^−3^ dilution of the enzyme preparation in BSII buffer, 70 µl of BSII buffer and 100 µl of 1:50 Furimazine:NanoGlow assay buffer. Determine signal intensity using a Tristar plate reader luminometer (Berthold Technologies GmbH & Co. Kg, Bad Wildbad, Germany) with a 1 min delay and 1.5 s integration time.

### Generation of Gateway binary vectors for expression of C-terminal NanoLUC fusions in plants

Binary plant transformation vectors pGWB604, pGWB605, pGWB601, pGWB701 and derivatives pGWB634, pGWB635, pGWB636, pGWB737 carrying LUC+ for C-terminal translational fusions were kindly donated by [26, 27]. The plasmids were propagated in *E. coli* One Shot™ *ccd*B Survival™ 2 T1^R^ cells (Invitrogen). We created a series of vectors named pGWB60xNL3F10H, equivalent to the pGWB63x series, using the NL3F10H reporter instead of LUC+. This was achieved by amplifying a sub-fragment of the Gateway cassette and fusing it with NL3F10H and pGWB601 digested with NcoI/SacI by Gibson assembly. The CaMV35S (35S) promoter was amplified from vector pCAMBIA1305.1 (Genbank accession no AF354045, Cambia Labs), adding attB1 and attB2 sites and recombined into pDONR221 (Invitrogen) using Gateway BP Clonase (Invitrogen). After sequence verification the CaM35S promoter was introduced into pGWB601NL3F10H and pGWB635 (LUC+) by a Gateway LR Clonase (Invitrogen). The genomic sequence coding for *BOA* from − 1000 to + 1053 (*BOAp:BOA*) without the terminal stop codon was amplified from Col-0 genomic DNA purified using DNeasy Plant Mini kit (Qiagen) and cloned into pGWB601:NL3F10H and pGWB635 (LUC+), by a BP Clonase recombination.

### Plant transformation and selection of homozygous lines

Parental Arabidopsis plants of accession Col-0 or Col-0 CCA1p:LUC+ (Millar Lab stock #G0129) were stratified for 2 days at 4 °C then transferred to 16L:8D at ~ 100 µmol m^−2^ s^−1^ from warm white fluorescent light bulbs. After 1 month in these conditions, plants were decapitated to promote branching and dipped in a culture of *Agrobacterium tumefaciens* ABI strain transformed using chemical competence and liquid nitrogen using the constructs described above. The ABI strain was kindly provided by Prof. Seth Davis (The University of York, UK). T1 seed was collected and primary transformants selected by BASTA resistance. The collection was then screened for 3:1 segregation of BASTA resistant:sensitive T2 generation seedlings on solid ROBUST media [1% Agar, 1/2 MS (Murashige and Skoog basal salt mixture, Duchefa, Haarlem, The Netherlands), pH 5.8 NaOH adjusted], supplemented with 10 µg/ml Bialaphos (Sigma). Lines with segregation consistent with a single locus of transgene insertion were taken to homozygosity by selecting on Levington F2 + S compost (ICL Everris Limited, Ipswich, UK) for segregants with 100% Bialaphos resistant T3 progeny. All other studies used T3 plants.

### Plant material and growth conditions for luciferase *in planta* assays

For imaging experiments with FLUC and NanoLUC, seeds were surface sterilised and individual seeds placed in flat-bottomed, white 96-well plates (LUMITRAC; Greiner) containing 150 µl of solid ROBUST media. The plate containing seeds was stratified for 48 h at 4 °C, then transferred to entrainment conditions (12L:12D, 21 °C, ~ 100 µmol m^−2^ s^−1^ cool white fluorescent light) in a controlled environment cabinet. After 1 week, 50 µl of either a 1:50 dilution of Furimazine (Promega) with 0.01% Triton X-100 (for NanoLUC) or 5 mM Luciferin (Sigma-Aldrich) in 0.01% Triton X-100 (for FLUC) were added to each well. Plates were transferred to a 12L:12D photoperiod of 50 µmol m^−1^ s^−1^ of monochromatic red and blue LED light at 21 °C for 3 days, then the photoperiod was changed to constant light with the same LED light source mounted on the plate reader. Bioluminescence was measured during this period every 30 min with an integration time of 1.5 s per well using an automated Tristar luminometer plate reader.

### Computational resources and data analysis

Docker was used for ensuring reproducibility of computational results. Jupyter notebooks document the data analysis for all plots in this work. BOA transgenic lines Period analysis was performed using FFT-NLS and plotted in the Biodare2 webservice [17].

## Data Availability

Biological material, raw data and data-analysis scripts are available from the authors upon reasonable request.
